# Effects of Natural Sorbents on the Germination and Early Growth of Grasses on Soils Contaminated by Potentially Toxic Elements

**DOI:** 10.3390/plants9111591

**Published:** 2020-11-17

**Authors:** Ingrid Turisová, Tatiana Kviatková, Katarzyna Możdżeń, Beata Barabasz-Krasny

**Affiliations:** 1 Department of Biology and Ecology, Faculty of Natural Sciences, Matej Bel University in Banska Bystrica, Tajovského 40, 974 01 Banská Bystrica, Slovakia; tatiana.kviatkova@umb.sk; 2 Institute of Biology, Pedagogical University of Krakow, Podchorążych 2 St., 30-084 Kraków, Poland; katarzyna.mozdzen@up.krakow.pl (K.M.); beata.barabasz-krasny@up.krakow.pl (B.B.-K.)

**Keywords:** grasses, mining heaps, natural sorbents, potentially toxic elements, remediation

## Abstract

The reclamation of abandoned mining heaps rich in potentially toxic elements (PTEs) is critical for the environment. We carried out a laboratory experiment studying the effects of the addition of four natural sorbents (biochar, bentonite, chicken manure and organo-zeolitic substrate) to soils contaminated with PTEs, predominantly Cu, As and Sb, on the germination and growth of the autochthonous grasses *Agrostis capillaris, A. stolonifera, Festuca rubra* and *Poa pratensis*. The experiment used Petri dish tests with water extracts of contaminated soil and soil neutralised with the four sorbents. Standard indexes of the germination process were used (germination percentage, time required for 50% germination index, speed of emergence), and different values were found depending on the plant species and sorbent used. However, the percentage of seeds germinating was lower for each sorbent compared to the control (distilled water). The fresh mass values were positively stimulated by all sorbents. Electrolyte leakage was the highest in seedlings watered with an extract of untreated soil from the heap compared to extracts from treated soils and the control. This can be interpreted as eliminating the harmful effects of increased potentially toxic element (PTE) contents by sorbents, which can be useful in remediation processes.

## 1. Introduction

Extreme habitats include areas once occupied by the mining industry, which left behind mine heaps and various types of toxic waste. These habitats have very specific plant cover because they are often characterised by a lack of soil and nutrients (including humus) and often a constant lack of moisture [1,2]. An additional problem is the fact that various types of toxic compounds, including many potentially toxic elements (PTEs), remain in the ground on mining heaps. Although many PTEs are indispensable for plants, they can block their development at higher concentrations. This negative effect depends on the PTE’s bioavailability in soil solutions, which is influenced by pH, organic matter content, microbial activity and cation exchange capacity of the soil. The contents of toxic substances is a colonisation barrier for many species of plants, even those with a relatively wide ecological scale [3,4,5,6].

However, there are plants that can tolerate this kind of extreme habitat. They usually colonise small depressions in the mining heap or sites with decaying material in the substrate that are relatively wet. Dead organic matter can gradually build up between stones, which will form humus over time if other unfavourable factors such as surface runoff are avoided. As a result, this can lead to the formation of a specific mosaic of vegetation. According to previously conducted studies of mining areas, perennial plants predominate in the heaps, while annual and biennial plants are relatively rare [7,8,9]. Heaps with untypical metal contents in the substrate are like ecological islands, as they can only be inhabited by species with specific physiological adaptations. In general, they do not differ morphologically from species growing under more favourable conditions, but their physiology is different. These adaptations are the result of evolutionary processes to which this small group of plants was exposed, enabling them to exist in phytotoxic conditions [10,11].

Because of the unusual conditions in mine heaps, plant succession results in new and unique plant communities [12,13,14]. During colonisation, lichens and bryophytes appear on the surfaces of stones and rocks and between the stones, grasses and tolerant dicotyledon species [12,13]. As the heaps usually originated from different periods of mining, the vegetation formed on them occurs at different stages of succession [7,15]. However, the spontaneous succession processes are strongly stretched in time, and there is a great need for the rehabilitation of mining heaps. This would contribute to faster colonisation of these areas and thus reduce the flow of toxic elements to the neighbouring areas. Therefore, studies on the tolerance of common plant species to post-mining soils containing specific components are needed. Another significant problem is the binding of toxins present in the substrate by the inhabited species. However, studies on this issue are generally lacking.

The aim of our experiment was to investigate the effect of the addition of four natural sorbents (biochar, bentonite, chicken manure and organo-zeolitic substrate) to soils contaminated predominantly with copper (Cu) on the germination and early growth of four common grass species, *Agrostis capillaris* L., *A*. *stolonifera* L., *Festuca rubra* L., *Poa pratensis* L. The use of sorbents is proving to be an economically and environmentally-friendly form of reducing the content and bioavailability of PTEs in contaminated soils [16,17,18]. Biochar is caused by a decrease in bioavailable fractions of Cd, Cu, Pb and Zn [19], while bentonite Cd, Pb, Zn [20] and chicken manure reduces the content of Pb, Cd, Cr, As [21]. Perlite can sorb Pb, Cu, Ni, Cd [22], calcium carbonate (CaCO_3_), Pb, Ni and Cd [23]. Adding organo-zeolitic substrate to the soil not only improves the soil texture, but it also acts as a supplement of P, N, Ca and K nutrient elements [24]. Sorbents also have a positive effect on plant growth and a significant increase in biomass yield. Chicken manure was effective for *Trifolium pratense* L., *Dactylis glomerata* L., *Lolium perenne* L., *Elytrigia repens* (L.) Nevski [25], biochar *Triticum aestivum* L. [19], bentonite for *Oryza sativa* L. [26].

We asked the following questions: (i) Does the addition of sorbents have a positive effect on the germination parameters of the tested species? (ii) do sorbents induce a higher increase in the length of seedlings? (iii) do young seedlings achieve higher values of biomass and water content in cells thanks to sorbents? and (iv) does the addition of sorbents reduce environmental stress, reducing electrolyte leakage from the cells of the analysed grasses?

## 2. Results

### 2.1. Soil Analyses

The results of soil analyses are presented in Table 1. The copper content exceeds the European Union limit by almost 10 times [27]. The average pH of anthrosol (5.17) indicates strongly acidic soil.

### 2.2. Germination of Seeds

**Figure 1:**
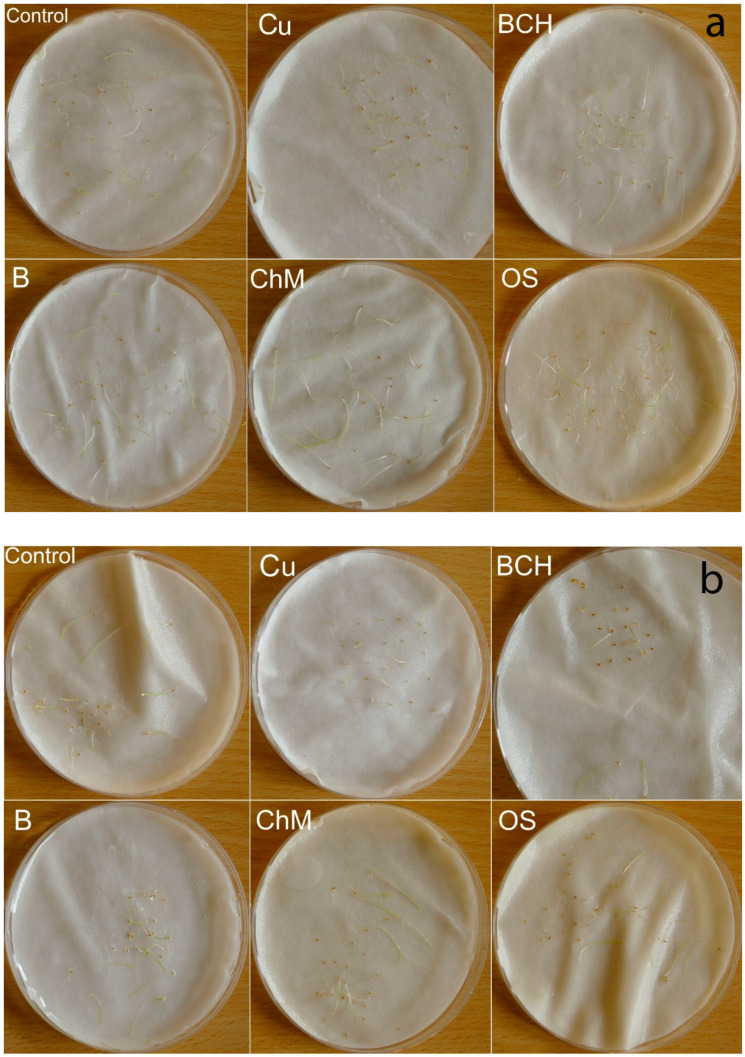

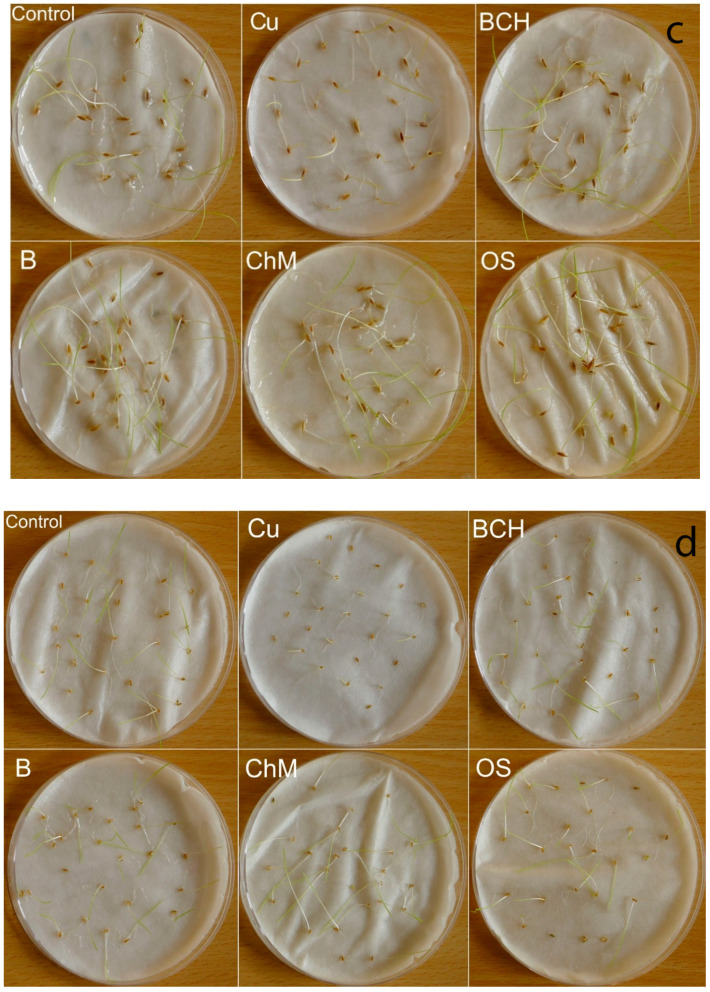
Petri dishes tests after 7 days of the experiment; (**a**) *Agrostis capillaris*, (**b**) *A. stolonifera*, (**c**) *Festuca rubra*, (**d**) *Poa pratensis*; Control—distilled water, Cu—copper soil; copper soil with sorbents: BCH—biochar, B—bentonite, ChM—chicken manure, OS—organo-zeolitic substrate.

On the third day of germination, all water extracts inhibited the germination of seeds of the tested species except for *Agrostis capillaris*, compared to controls. On the seventh day of germination, the germination percentage (GP) was generally higher for seeds watered with distilled water than for extracts. The exception was for *Poa pratensis*, for which the GPs were clearly higher than for the seeds from the control (Table 2). After the seventh day of the experiment, *Festuca pratensis* and *Poa pratensis* had germinated the best (Figure 1); the other two species had a slightly weaker development, regardless of the type of sorbent, but still better than on the soil contaminated with copper and other metals.

The values of the T50 index were similar, regardless of the species and type of extract used (including the control). Statistical analysis showed significant differences only between *A. stolonifera* and the other three studied species (Table 3).

**Figure 2:**
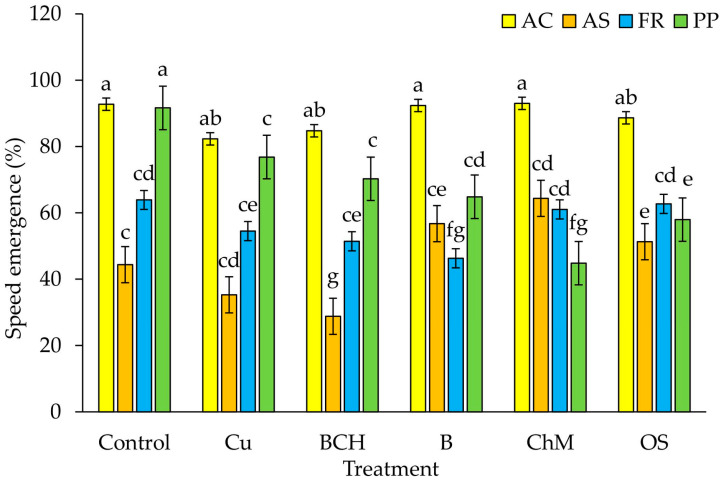
Speed of emergence (SE) seeds germinated on water extracts from contaminated soil; species: AC—*Agrostis capillaris*, AS—*A. stolonifera*, FR—*Festuca rubra*, PP—*Poa pratensis*; Control—distilled water, Cu—copper soil; copper soil with sorbents: BCH—biochar, B—bentonite, ChM—chicken manure, OS—organo-zeolitic substrate; mean ± SD (*n* = 5) different letters differ significantly according to Fisher’s test *p* ≤ 0.05.

The speed of emergence (SE) index in *A. capillaris* seedlings was not significantly different between the control and the soil extracts. In *A. stolonifera*, the SE was lowest in seedlings watered with BCH extracts, and highest in ChM, compared to the control. For *F. rubra*, similar SE values were found in the control and for ChM and OS extracts. The lowest SE values were observed for seedlings watered with B. In *P. pratensis,* the SE index was lowest in seedlings grown on the ChM extract and highest in the control (Figure 2).

Biometric analysis of seedlings treated with water soil extracts showed a positive effect of sorbents on elongation growth (Table 4). Regardless of species, B and OS stimulated elongation growth relative to seedlings from the control sample. The use of the manure sorbent (ChM) also stimulated elongation growth for *A. stolonifera* and *F. rubra*. Compared to the control, only the addition of carbon (BCH) had a negative effect on the growth of all tested seedlings.

The fresh mass of seedlings of the tested grass species on water extracts of soils with sorbents was higher than in the control (Table 5). Each of the water extracts had a positive effect on this parameter. The highest increase in fresh mass was observed for *A*. *capillaris* seedlings germinated on the OS extract. The lowest values of fresh mass relative to the control were found for *F*. *rubra* watered with the ChM extract. The dry mass of seedlings watered with soil water extracts was significantly higher in *A*. *capillaris* and *P*. *pratensis* than controls. In the case of *A*. *stolonifera* and *F*. *rubra*, all extracts significantly reduced the dry mass of seedlings. Tissue water content values of the seedlings were generally similar between the control and the tested soil extracts. Regardless of the species, the highest increase in the percentage of water content was found in seedlings watered with OS, ChM and B, compared to the control.

**Figure 3:**
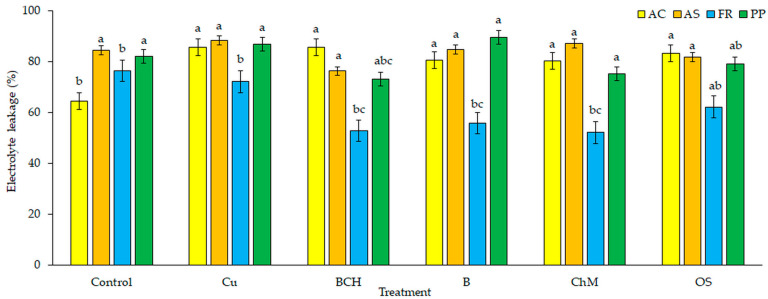
Electrolyte leakage (EL) in seedlings treated with water extracts from different soils; species: AC—*Agrostis capillaris*, AS—*A. stolonifera*, FR—*Festuca rubra*, PP—*Poa pratensis*; Control—distilled water, Cu—copper soil; copper soil with sorbents: BCH—biochar, B—bentonite, ChM—chicken manure, OS—organo-zeolitic substrate; mean ± SD (*n* = 5) different letters differ significantly according to Fisher’s test *p* ≤ 0.05.

The percentage of electrolyte leakage of *A. capillaris* seedlings was higher in each of the tested solutions than in the control (Figure 3). The highest degree of water-ion balance destabilisation was found for seedlings germinated from seeds watered with Cu and BCH extracts. For the remaining three extracts, there was a reduction in electrolyte leakage disturbance, but it was still significantly higher than in seedlings watered with distilled water. For *A. stolonifera*, electrolyte leakage was similar for soil extracts and the control, with no significant differences found. In the case of seedlings of *F. rubra*, there were significant decreases in the degree of electrolyte leakage for all water extracts except for seedlings watered with Cu extracts, which were similar to the control. Only slight changes were found in electrolyte leakage for *P. pratensis* seedlings germinated on BCH and ChM extracts compared to the control and other soil extracts.

The sources data for pH, germination index, time required for 50% germination index, speed of emergence, length whole seedlings and inhibition of percentage (IP), fresh masses, dry masses and tissue water content, electrolyte leakage are given in Table S1.

## 3. Discussion

**Figure 4:**
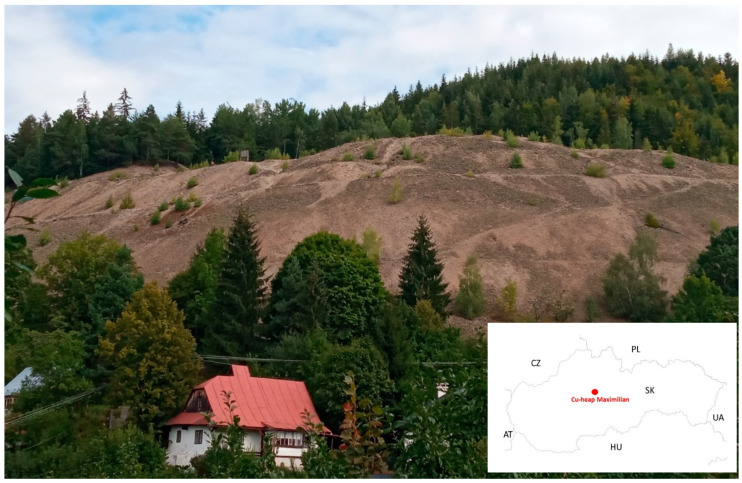
The copper mine heap of Maximilián in Špania Dolina village, and showing the location in Slovakia (Photo T. Kviatková).

Copper is a micronutrient that plants need, but only in small amounts. It is a component of proteins and enzymes and as such plays an important role in the processes of photosynthesis and respiration, and also participates in the lignification and growth of plants. Without copper, the leaves of plants twist and turn dark green. At low concentrations, it has a stimulating effect, but at high concentrations, it inhibits both seed germination and plant growth, and in extreme cases, leads to complete necrosis [28,29,30,31,32,33]. As mentioned previously, in mining areas, copper deposits are generally accompanied by heavy metals [34], resulting in many negative environmental effects. These heavy metals reduce the productivity of plants and pose a threat to entire ecosystems [35] (Figure 4). They cause environmental stress, influencing biochemical, genetic and physiological changes in plants [36,37]. However, plants have developed potential mechanisms to combat heavy metal toxicity problems. For instance, they produce low molecular mass thiols that have a high affinity for toxic metals [38]. The most important biologically active thiols in this respect are glutathione (GSH) and cysteine. GSH is a substrate for the synthesis of phytochelatin and is of key importance for the detoxification of heavy metals such as cadmium or nickel [39]. Phytochelatins form complexes with toxic metal ions in the cytosol and then transfer them to the vacuole [40]. In this way, they protect plants against the harmful effects of heavy metals, though it is likely that these mechanisms work at different levels in different plant species. According to [41], Cu, As and Sb are the most important toxic metals in terms of environmental risk in the studied locality and pose a serious danger due to the highly toxic nature of their compounds. Already in our research, the contents in the soil are very high, but [42] found even higher (Cu 386–4630, As 37–983, Sb 46.3–1403 in mg·kg^−1^).

**Figure 5:**
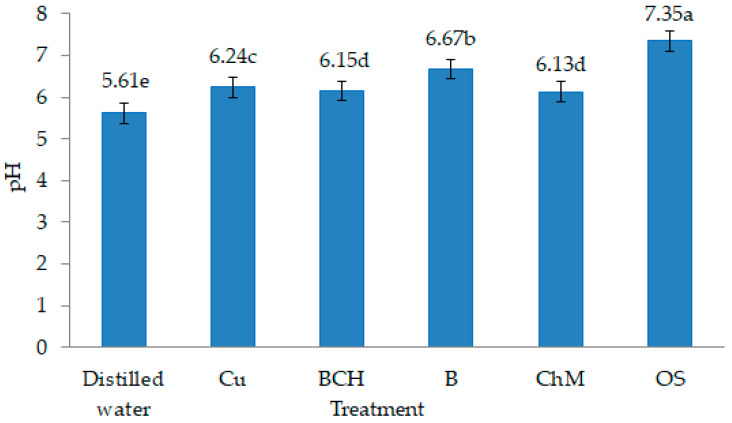
pH values of water soil extracts: Control—distilled water, Cu—copper soil; copper soil with the sorbents: BCH—biochar, B—bentonite, ChM—chicken manure, OS—organic substrates; mean ± SD (*n* = 5) different letters differ significantly according to Fisher’s test *p* ≤ 0.05.

The availability of heavy metals in soils is influenced by the presence of organic matter and soil pH [43]. In neutral and slightly alkaline soil solutions, Cu is present in the form of inorganic complexes [44]. The high content of organic matter and clay minerals reduces the bioavailability of metals while increasing their fraction in the soil [45,46]. pH values > 6.5 reduce the amount of readily soluble forms of metals in the soil and limit their uptake and accumulation by plants [47]. In our study, water extracts with sorbents were characterised by higher pH values compared to non-modified soil extracts contaminated with copper and other metals (Figure 5). Therefore, it can be concluded that the natural sorbents added to the soil probably affected the immobilisation of heavy metals [48].

Germination is one of the first stages of seed contact with a stress factor, making it a kind of sensitivity index and measure of tolerance to chemical and physical conditions of the rhizosphere [49,50,51]. During germination, the phytotoxicity of Cu and accompanying heavy metals depends on their concentration levels [52,53,54] (Figure 1). Excesses cause oxidative stress by activating reactive oxygen species and inhibiting catalase activity [55]. Under stress, plant germination and growth are slower as the plant produces smaller cells with thicker cell walls [56], with the germination capacity of seeds reduced with increasing concentrations [57,58]. One of the toxic properties of these elements may be due to a shortage of water in the extracts, which inhibits cell expansion and reduces carbon uptake [59].

The germination percentage (GP) index in our study was higher for seeds watered with distilled water than with extracts from contaminated soil and sorbents (Table 2). The T50 index was significantly different only for *Agrostis stolonifera*, compared to the other three analysed species (Table 3). However, the values of the speed of emergence (SE) index changed depending on the species and the extract used (Figure 2). These different reactions of the tested seeds to the water extracts with sorbents used may have resulted from their differing protective properties against stress. Seeds are equipped with sensing mechanisms thus that germination will only take place when environmental factors are favourable. In addition to complexes with phytochelatins, plant defence strategies in the fight against heavy metals include reduced absorption by formation, activation of osmolytes substances and antioxidants and changes in enzyme expression [60]. Another protective barrier is the seed coat, which limits the penetration of harmful substances into the seed [33,61]. This is probably related to our observations after the seventh day of the experiment: *Festuca pratensis* and *Poa pratensis*, with larger seeds and a thicker seed coat, germinated and developed better (Figure 1).

According to [62], the stage of seedling growth and development is more sensitive to metals than germination. The toxic effects of copper on plant growth have already been confirmed, e.g., for mustard [63], wheat [31], and pine [64]. In our study, the biometric analysis of seedlings showed a stimulating effect of OS extracts and an inhibitory effect of the addition of carbon (BCH) on the growth of all tested species (Table 4). The inhibition of seedling growth most likely resulted from the impairment of the basic seed functions. Copper and heavy metals present in toxic amounts modify the ultrastructure of the cell and disturb its homeostasis. They affect cell nuclei and cell division, causing a reduction in mitotic activity, cytokinesis disturbances, DNA and RNA damage, decreased transcription activity, chromatin condensation, chromosomal aberrations, destruction of the nuclear envelope, reduction of the volume of nuclei and an increase in the number of micronuclei [65]. All these mechanisms ultimately impair plant growth and development.

The effects of heavy metal toxicity on plant mass also depend on their concentrations [66,67]. Our results showed that each of the water extracts with sorbents had a positive effect on the values of the fresh mass of grass species. Seedling dry mass was significantly higher in *Agrostis capillaris* and *P. pratensis*, while for *A. stolonifera* and *F. pratensis*, an inhibition of mass gain was seen (Table 5; Figure 1). Copper induces the mobilisation of biomass by releasing glucose and fructose, inhibits the decomposition of starch and sucrose in tissues by limiting the activity of alpha-amylase and invertase isoenzymes [55]. Its excess is related to the ability to accumulate free amino acids, e.g., proline and glycine [68,69]. A study by Boroş et al. [56] explained the increase in dry mass by the capturing of metal ions that bind to the cell wall, creating a heavier cell. Reductions of seedling biomass may also be due to limitations in protein formation and disturbances in carbohydrate translocation [70,71]. Thus, copper affects the overall metabolism, water uptake, and, depending on the concentration, limits the synthesis of plant nutrients [72].

In plants, stress leads to anatomical, morphological and physiological changes in root tissues, causing an inhibition of the root’s water and ion transport functions. The specific surface area of the roots depends on the number and size of intercellular spaces and the properties of the cell walls. Monocotyledonous plants have a lower cation-exchange capacity and take up polyvalent ions to a lesser extent. In the case of copper, they show a weaker ability to immobilise it in the cell wall [73]. Limitations on water uptake by plants reduce their turgor, which leads to limits in elongation growth, and the inhibition of root growth is an additional cause of limited water uptake [74]. Our study found the highest values of tissue water content for seedlings watered with OS, ChM and B, compared to the control (Table 5). This demonstrates a neutralisation of the toxic properties of copper on the physiology of seedlings of the studied grass species and a positive effect of natural sorbents.

Cell membranes, which are complex and dynamic structures, participate in the regulation of water transport. They play an important role in the resistance of plant cells to environmental stresses [64]. They constitute selective permeability barriers that regulate the molecular and ionic composition of the intracellular environment through specific channels, conveyors and pumps. Undamaged cell membranes allow water molecules to enter the cell interior and are barriers for molecules of substances dissolved in the cell sap. Copper and heavy metals contribute to changes in the permeability properties of cell membranes. Due to lipid peroxidation, they disrupt their function and structure [75,76]. They can also displace basic metal ions, which usually leads to a reduction or loss of enzymatic activity, the generation and accumulation of reactive oxygen species, and even programmed cell death [77,78]. In our experiment, the percentage of electrolyte leakage was the highest in seedlings watered with Cu soil extracts without any sorbents added. Regardless of the species, the addition of natural sorbents had a positive effect on the membranous structures of cells (Figure 3). This indicates that the influence of copper on changes in the structures of cell membranes is limited, and thus the loss of semi-permeable properties is reduced.

In summary, in our experiment, developmental changes in seedlings most likely resulted from disturbances arising at the stage of embryogenesis and were the result of the direct contact of seeds with extracts, which to a varying degree, eliminated the harmful effects of toxic metals. Further physiological studies of various plant species growing under toxic conditions will allow us to better understand their biochemistry, learn the details of defence strategies and ways to overcome stress, which at the same time will contribute to increasing their productivity. This will enable the identification of species resistant to pollution and their use in the more rapid restoration of vegetation in highly contaminated areas.

## 4. Materials and Methods

### 4.1. Study Area

The soil for testing was taken from the Maximilián heap (48°48′29″ N, 19°07′59″ E), located in the village of Špania Dolina (Central Slovakia; Figure 4). The area of the Špania Dolina ore deposit was one of the most recognisable copper deposits in the history of mining in Europe, with the mineralisation creating a vein 4 km long and 1.5 km wide [79]. The first written reports of ore mining in the area of Staré Hory and Špania Dolina date back to the 11th century (from 1006), although the nearby Piesky deposit was probably already exploited in the Late Stone Age [80,81]. The maximum extraction of copper ore, along with its extremely valuable silver content, took place in the years 1496–1546 (58,234 t Cu, 111,280 kg Ag). From the 17th century onwards, mining gradually decreased until it completely disappeared in the early 20th century [82].

In the Maximilián heap, the anthrosol remnants of the mining activity were contaminated by a high content of PTEs. Most Cu comes from chalcopyrite (CuFeS_2_), and As and Sb from tetrahedrite. The vegetation cover of the heap was very sporadic and sparse, with growth and development made difficult by an excess of coarse-grained tailings, a lack of soil matrix, water, nutrients, increased contents of PTEs, and other environmental and ecological factors.

### 4.2. Seeds Material with Short Characteristic of Species

Four common grass species typical for Eurasian areas were selected for the experiment: *Agrostis capillaris* L., *A. stolonifera* L., *Festuca rubra* L., *Poa pratensis* L. Of these, *P. pratensis* had the highest utility value as a forage grass, while the other species were of lesser importance for forage [83]. However, they were suitable for land reclamation, creating dense turfs and grasslands. For this experiment, the seeds were bought from DLF SeeDs (Hladké Životice, Czech Republic).

All 4 species were certainly native to most of Eurasia [84,85]. They can be found growing in a variety of habitats, including grasslands and meadows, wetlands, fields, roadsides, forest edges, etc., and as a pioneer species on disturbed sites [86]. *Agrostis capillaris*, *A. stolonifera* and *Festuca rubra* were considered as facultative metallophytes [87]. *Agrostis capillaris* and *Poa pratensis* were also used in erosion control due to its dense, vigorous turf forming habitat [88].

### 4.3. Soil Analysis and Modification

The 3 soil samples with the weight of 2–3 g were analysed in the Geochemical and Assaying Laboratories of Bureau Veritas Commodities Canada Ltd. in Vancouver, by inductively coupled plasma emission spectrometry (ICP-ES) method. Samples were digested in aqua regia and acid digestion by HNO_3_, HClO_4_ and HF heated until dryness, to decompose most minerals, including silicates to metal salts. The residues were dissolved in concentrated HCl. 

The contaminated soil from heap Maximilián was subsequently mixed with natural sorbents, which were able to immobilize PTEs. Water extracts were prepared from soil mixtures (Table 6).

### 4.4. Water Extract and pH Values

100 g of each type of soil treatment was flooded with 150 mL of distilled water, thoroughly mixed, wrapped in aluminium foil, and put on a shaker for 2 h. The water solutions were left at room temperature for 24 h in order to extract the chemical substances they contained. Then, the water soil solutions were filtered through filter paper and stored in a refrigerator for the duration of the experiment. pH values of each of the solutions were determined (pH-meter CX-701, Elmetron, Zabrze, Poland), and the pH of water extracts of soils with the addition of natural sorbents ranged from 6.15 to 7.35. The highest values were for OS, and the lowest for BCH (Figure 5).

### 4.5. Germination Conditions

Grass seeds (each separately) were sterilised in 1% acetone for 5 min, then rinsed 3 times with distilled water. 25 seeds of each species were placed on separate sterilised Petri dishes (Ø 9 cm) with 3 layers of Whatman filter paper (Grade 1:11 μm—medium flow filter paper). On the first day, seeds were watered with 5 mL of water soil extracts. Every other day, the seeds on Petri dishes were wetted with 3 mL of each extract, respectively. The control group consisted of seeds watered with distilled water. For the duration of the experiment, seeds were placed in the dark, at room temperature. After 3 and 7 days, seeds were counted. Germinated seeds were considered those whose germinal root was equal to half the size of the seed. A germination experiment was performed in 3 repetitions for each of the extracts and control samples.

### 4.6. Germination Parameters

The effect on the germination capacity of seeds was evaluated by the germination percentage—GP [89], the time required for 50% germination—T50 [90], and speed of emergence—SE [91] according Table 7.

### 4.7. Biometric Analysis

The effect of water extracts on seedlings length was determined using a caliper (Topex 31C615, Grupa Topex, Warszawa, Poland), with an accuracy of 1 mm. The inhibition percentage (IP) expressed as a percentage of control seedlings was measured according to Islam et al. [92].

IP = (1 − (L_E_/L_C_)) × 100 (1)

where: L_E_—seedling length (cm) treated with the aqueous extract, L_C_—seedling length (cm) treated with the distilled water (control group). Inhibition percentage (IP) of growth (expressed as control %)—a minus (−) value indicates an increase of seedlings length, and a plus (+) value indicates a decrease of elongation of seedlings.

### 4.8. Fresh and Dry Mass and Tissue Water Content

Fresh mass (FM) of seedlings was determined on a scale (Ohaus Adventurer Pro, OHAUS CORPORATION, NJ, USA). The plant material was dried for 48 h at 105 °C in a dryer (WAMED SUP 10, WAMED Wytwórnia Aparatury Medycznej SSP, Warszawa, Poland) to obtain the dry mass (DM). The total water content was determined according to the formula:

H_2_O (%) = 100 − ((DM × 100)/FM), (2)

### 4.9. Electrolyte Leakage

Electrolyte leakage was measured in 7 days of seeds germination according to the method used by [64]. A single grass seedling was placed in polypropylene vials with 30 mL distilled water and shaken for 3 h on a shaker (Labnet International Inc., Edison, NJ, USA) to determine the leakage of the electrolyte from live cells (E1) by conductivity meter CX-701 (Elmetron, Zabrze, Poland) with the electrode (K = 1.02) (Elmetron, Zabrze, Poland). Then plant material was frozen at −75 °C to macerate the tissues. After 24 h, the seedling in water was defrosted and subjected to the same procedure (E2) as samples with live material. On the basis of the results, the total electrolyte leakage was determined according to the formula:

EL (%) = (E1/E2) × 100(3)

### 4.10. Statistical Analysis

Statistical analysis was performed using the multivariate-way ANOVA analysis of variance test. The obtained results were subjected to multivariate analysis examined the relationship between a given parameter and species and extracts. At the same time, all species were analysed in order to verify how they respond to stress factors in the form of water extracts from soil. Which species was the least and which was the most sensitive. The differences between the means ± SD (*n* = 5) were measured by Fisher’s test at *p* ≤ 0.05 in the program StatSoft, Inc. 2018 (Warszawa, Poland). STATISTICA (data analysis software system), version 13.1.

## 5. Conclusions

Sorbents, used as an additive to neutralise copper (Cu) and other PTEs in soils, have a significant effect on the germination and early growth of grass seeds. Reactions seeds on soil water extracts depend on grass species and type of extracts. In general, the addition of any of the sorbents to the contaminated soil is beneficial. Soils with B and ChM had the most positive effect on seeds germination percentage (GP) (i).

In the case of the elongation growth of seedlings, a negative effect of BCH extracts and a positive effect of OS extract were demonstrated for all seed species (ii).

The fresh mass values were stimulated by all sorbent extracts. The dry mass of seedlings was higher in *Agrostis capillaris* and *Poa pratensis*, and lower in *A. stolonifera* and *Festuca rubra*. The tissue water content of the tested seedlings was higher for those grown with OS, ChM and B sorbents, compared to the control (iii).

Electrolyte leakage was highest in seedlings watered with Cu soil extracts for *Agrostis capillaris*. For the other studied species, although a decrease in the degree of destabilisation of cell membranes was observed, significant differences in the values of this parameter were not found compared to the control (iv).

The experiment confirmed the hypothesis that the addition of natural sorbents (especially organic substrates, chicken manure and bentonite) eliminates the toxic properties of copper and heavy metals contained in the soil, which is an important conclusion from the point of view of the reclamation of copper heaps. Sorbents have a positive effect on germination and early seed growth and reduce the destabilisation of seedling cell membranes. However, differences in plant responses to extracts depend on the species and the modifications applied to the extracts.

## Figures and Tables

**Table 1:** Content of potentially toxic elements (PTEs) (in mg·kg^−1^) and pH in contaminated soil.

PTEs (mg·kg^−1^)	Cu	As	Sb	Ag	Co	Pb	Zn	Ni	Al	pH
1st sample	1179	275	405	3.1	28	26	38	25	7.53	5.10
2nd sample	1017	273	390	2.7	27	26	39	24	8.58	5.16
3rd sample	1101	255	367	2.4	24	23	36	22	7.15	5.25
Average	1099	267.67	387.33	2.73	26.33	25	37.67	23.67	7.75	5.17

**Table 2:** Germination percentage (GP) index (%) after 3 and 7 days seeds germinated on water extracts from contaminated soil.

Treatment	Species							
	AC		AS		FR		PP	
	3	7	3	7	3	7	3	7
Control	73.6 ab ± 8.29	89.6 a ± 2.19	20.2 cf ± 7.16	41.6 c ± 9.21	57.6 cd ± 19.72	89.6 a ± 7.16	72.0 b ± 11.45	79.2 ab ± 4.50
Cu	77.6 ab ± 2.19	84.0 b ± 6.32	18.4 e ± 10.81	31.2 d ± 13.68	48.8 c ± 26.14	88.0 ab ± 6.32	70.4 b ± 16.15	92.0 a ± 4.90
BCH	76.0 ab ± 11.66	89.6 a ± 8.29	7.2 efg ± 5.93	23.2 d ± 3.35	43.2 c ± 7.16	84.0 b ± 4.00	64.0 c ± 14.14	90.4 a ± 8.29
B	76.0 ab ± 6.32	82.4 b ± 7.80	17.6 f ± 11.52	29.6 d ± 7.27	40.0 c ± 6.32	86.4 b ± 3.58	60.0 c ± 17.20	92.8 a ± 5.93
ChM	80.8 a ± 3.35	87.2 ab ± 6.57	17.6 f ± 7.80	27.2 d ± 9.12	54.4 cd ± 11.52	88.8 a ± 3.35	40.0 c ± 9.38	89.6 a ± 6.07
OS	80.8 a ± 9.55	91.2 a ± 4.38	13.6 f ± 8.29	25.6 d ± 7.27	50.4 cd ± 10.81	80.0 b ± 4.90	54.40 cd ± 11.52	93.6 a ± 4.56

AC—*Agrostis capillaris*, AS—*A. stolonifera*, FR—*Festuca rubra*, PP—*Poa pratensis*; Control—distilled water, Cu—copper soil; copper soil with sorbents: BCH—biochar, B—bentonite, ChM—chicken manure, OS—organic substrates; mean ± SD (*n* = 5) different letters differ significantly according to Fisher test *p* ≤ 0.05.

**Table 3:** The time required for 50% germination index (T50) seeds germinated on water extracts from contaminated soil.

Treatment	Species			
	AC	AS	FR	PP
Control	0.451 a ± 0.01	0.374 b ± 0.03	0.450 a ± 0.01	0.442 a ± 0.01
Cu	0.447 a ± 0.01	0.175 cd ± 0.38	0.450 a ± 0.01	0.452 a ± 0.01
BCH	0.451 a ± 0.01	0.227 bc ± 0.06	0.447 a ± 0.01	0.451 a ± 0.01
B	0.446 a ± 0.01	0.295 bc ± 0.08	0.449 a ± 0.01	0.453 a ± 0.01
ChM	0.449 a ± 0.01	0.236 bc ± 0.15	0.450 a ± 0.01	0.451 a ± 0.01
OS	0.452 a ± 0.01	0.231 bc ± 0.14	0.444 a ± 0.01	0.453 a ± 0.01

AC—*Agrostis capillaris*, AS—*A. stolonifera*, FR—*Festuca rubra*, PP—*Poa pratensis*; Control—distilled water, Cu—copper soil; copper soil with sorbents: BCH—biochar, B—bentonite, ChM—chicken manure, OS—organo-zeolitic substrate; mean ± SD (*n* = 5) different letters differ significantly according to Fisher’s test *p* ≤ 0.05.

**Table 4:** Lengths of whole seedlings and inhibition of percentage (IP) of seeds germinated on water extracts from contaminated soil.

Treatment	Species							
	AC		AS		FR		PP	
	(cm)	IP (%)	(cm)	IP (%)	(cm)	IP (%)	(cm)	IP (%)
Control	2.26 cde ± 0.60		1.62 f ± 0.54		5.05 bc ± 1.02		2.94 de ± 0.55	
Cu	2.69 de ± 0.56	−24.15	1.94 ef ± 0.79	−52.01	4.33 c ± 1.11	9.99	3.01 d ± 0.89	−4.54
BCH	1.99 def ± 0.51	6.56	1.47 ef ± 0.67	1.67	4.20 c ± 0.86	13.05	2.66 de ± 0.82	5.00
B	2.26 cde ± 0.39	−5.37	1.83 de ± 0.46	−34.55	4.99 bc ± 0.90	−1.54	3.11 d ± 0.66	−7.57
ChM	1.86 ef ± 0.49	12.00	2.53 ce ± 0.79	−73.01	6.08 a ± 1.04	−25.88	1.90 ef ± 0.68	34.69
OS	2.71 de ± 0.53	−28.10	2.06 def ± 0.75	−55.04	5.43 bc ± 1.10	−12.27	4.04 cd ± 0.75	−42.57

AC—*Agrostis capillaris*, AS—*A. stolonifera*, FR—*Festuca rubra*, PP—*Poa pratensis*; Control—distilled water, Cu—copper soil; copper soil with sorbents: BCH—biochar, B—bentonite, ChM—chicken manure, OS —organo-zeolitic substrate; IP—expressed as % of control—positive values indicate growth inhibition, negative values indicate seedling growth stimulation; mean ± SD (*n* = 5) different letters differ significantly according to Fisher’s test *p* ≤ 0.05.

**Table 5:** Fresh, dry masses expressed as % of control and total water content in seedlings treated with water extracts from different contaminated soils.

Treatment	Species			
	AC	AS	FR	PP
**Fresh Mass as % of Control**				
Cu	122.00 bd ± 0.01	205.45 b ± 0.01	165.21 cd ± 0.02	168.98 cd ± 0.01
BCH	128.00 bd ± 0.01	180.00 cd ± 0.01	166.44 cd ± 0.02	168.25 cd ± 0.01
B	124.00 bd ± 0.01	234.55 b ± 0.01	147.87 cd ± 0.01	181.02 cd ± 0.01
ChM	106.00 bcd ± 0.01	162.79 cd ± 0.01	104.61 bcd ± 0.01	165.73 cd ± 0.01
OS	364.00 a ± 0.05	170.91 bcd ± 0.01	160.29 cd ± 0.02	164.60 cd ± 0.01
**Dry Mass as % of Control**				
Cu	162.50 a ± 0.01	77.78 de ± 0.01	88.17 d ± 0.01	103.45 cd ± 0.01
BCH	137.50 c ± 0.01	61.11 f ± 0.01	97.63 cd ± 0.01	131.03 c ± 0.01
B	150.00 b ± 0.01	55.56 g ± 0.01	88.17 d ± 0.01	124.14 c ± 0.01
ChM	125.00 c ± 0.01	66.67 e ± 0.01	85.80 e ± 0.01	103.45 cd ± 0.01
OS	150.00 b ± 0.01	77.78 de ± 0.01	84.62 e ± 0.01	134.48 c ± 0.01
**Tissue Water Content** (**%**)				
Control	91.89 a ± 4.07	66.89 e ± 7.31	80.58 de ± 1.02	89.40 de ± 5.67
Cu	88.45 de ± 4.65	85.98 b ± 4.71	89.78 be ± 1.80	93.73 a ± 3.30
BCH	90.99 a ± 1.80	87.73 b ± 4.07	88.73 bc ± 0.98	91.67 a ± 0.52
B	89.96 b ± 1.65	91.40 a ± 3.40	88.69 bc ± 2.38	92.69 ab ± 0.22
ChM	90.42 a ± 3.57	85.19 c ± 9.57	89.36 bc ± 2.85	90.79 ab ± 3.07
OS	93.07 a ± 3.00	85.48 cde ± 3.65	89.96 b ± 2.85	91.25 ab ± 0.78

AC—*Agrostis capillaris*, AS—*A. stolonifera*, FR—*Festuca rubra*, PP—*Poa pratensis*; Control—distilled water, Cu—copper soil; copper soil with sorbents: BCH—biochar, B—bentonite, ChM—chicken manure, OS—organo-zeolitic substrate; mean ± SD (*n* = 5) different letters differ significantly according to Fisher’s test *p* ≤ 0.05.

**Table 6:** Soil modifications are used to prepare water extracts to experiment.

Abbreviation	Soil	BCH	B	ChM	OS
Full name	Cu contaminated soil from a mining heap	biochar	bentonite	chicken manure	organo-zeolitic substrate
Mixture proportions per 1 unit weight of soil		0.2—biochar	0.1—bentonite	0.01—chicken manure	0.5—perlite 0.3—CaCO_3_ 0.1—chicken manure
		0.8—soil	0.9—soil	0.99—soil	0.91—soil

**Table 7:** Formulas of germination indexes used in the experiment.

Germination Index	Formula	References
GP germination percentage	(number of germinated seeds each day/total number of seeds used in biotest) × 100	AOSA [89]
T50 time required for 50% germination	ti + ((N/2) − nj) × (ti − tj))/(ni − nj) where: N is the final number of germination and ni, nj cumulative numbers of seeds germinated by adjacent counts at times ti and tj when ni < N/2 < nj	Farooq et al. [90]
SE speed of emergence	(number of germinated seeds on the first day of germination/number of germinated seeds on the last day of germination) × 100	Islam et al. [91]

## Supplementary Materials

The following are available online at https://www.mdpi.com/2223-7747/9/11/1591/s1, Table S1: Sources data for (**a**) pH; (**b**) germination index (GI); (**c**) time required for 50% germination index (T50); (**d**) speed of emergence (SE); (**e**) length whole seedlings and inhibition of percentage (IP); (**f**) fresh masses, dry masses and tissue water content (TWC); (**g**) electrolyte leakage.
